# Obesity among parents and children from an indigenous rural community in Mexico

**DOI:** 10.1590/S1516-31802007000600014

**Published:** 2007-11-01

**Authors:** Ramón Arrizabalaga-Amarelo, Hugo Mendieta-Zerón

**Affiliations:** San Cristóbal Huichochitlán, Toluca, State of Mexico, Mexico

## Obesity in developing countries

Whereas the prevalence of obesity is increasing in both developed and developing countries,^1^ there are few data from rural and indigenous populations relating to cardiovascular disease and metabolic risk factors.^2^

We have explored the prevalence of overweight and obesity among parents and children of the predominant Otomí indigenous group in the community of San Cristóbal Huichochitlán, Toluca, State of Mexico, Mexico, excluding pregnant women. Statistical analysis was done using the Statistical Package for Social Science (SPSS) software, version 10 (SPSS Inc., Chicago, United States).

This study included 56 individuals: 22 individuals over 18 years of age and 34 younger individuals. All their families (10) had very low incomes (less than 0.5 United States dollars per hour of work). Three mothers and one father were illiterate, one mother and five fathers had not completed primary school, while four mothers and two fathers had completed their primary school education and one mother and one father had also attended secondary school. The other two individuals over 18 years were not neither fathers nor mothers but teenagers. Although seven of the ten families had their own houses, only eight had electricity and four had municipal water.

Furthermore, this Mexican indigenous group has genetic homogeneity and overexpressed polymorphisms that are associated with complex diseases.^3^ Because of the high emigration rate, these characteristics constitute a health problem in both Mexico and also the United States.

Among the adults, the mean age, weight, height and body mass index (BMI) were 32.63 ± 8.84 years, 63.59 ± 8.93 kg, 1.57 ± 0.09 m and 25.79 ± 3.1 kg/m^2^ respectively. The BMI (weight in kilograms divided by the square of the height in meters) that was taken as the cutoff for obesity was > 28.0 and > 27.0 and kg/m^2^ for men and women respectively, as suggested for a Mexican population.^4^ The mean systolic blood pressures were 125 ± 7.07 and 106.87 ± 9.23 mmHg and the mean diastolic blood pressures were 85 ± 7 and 66.25 ± 11.57 mmHg, for men and women respectively. The prevalence of obesity among men and women over 18 years was 27.27% and 45.45% respectively. Among the fathers it was 33.33% and among the mothers it was 44.44%, without statistically significant difference attributable to age (Mann-Whitney U test, p = 0.387).

Almost 100% of the adult females had a higher waist circumference than the cutoff value (> 80 cm). We found one boy and one girl in the 95^th^ percentile of the weight-to-age table, and there were eight undernourished children. Our findings among the adults were similar to other studies.^5^ In fact, it was alarming that in 66.66% of the families at least one parent was obese, but surprisingly we did not find any hypertensive women. Other negative characteristics in this community were insufficiencies of fruit and vegetables in the diet and declining consumption of traditional foods such as rice and beans. In addition to the two children in the 95^th^ percentile mentioned above, the prevalence of stunting among individuals less than 18 years of age was 23.52%.

The high prevalence of overweight and obesity in developing countries such as Mexico and their association with diseases such as type 2 diabetes mellitus, heart disease and cancer is likely to have made these pathological conditions a high-priority problem on the agenda of the Ministry of Health. Unfortunately, the nutritional deterioration has most likely been due to the recent increases in the prices of basic products such as milk, eggs and corn tortillas.

Finally, obesity prevention measures aimed towards children are needed urgently. We believe that the Mexican government should encourage body mass index monitoring during childhood, with subsequent interventions as necessary to prevent obesity and type 2 diabetes mellitus in adulthood, and also a scientifically based public school physical activity program.
